# Ablative radiotherapy in castration‐resistant prostate cancer

**DOI:** 10.1111/bju.70368

**Published:** 2026-07-02

**Authors:** Tobias Hölscher, Fabian Lohaus, Lydia Koi, Jörg Kotzerke, Sebastian Hoberück, Christian Thomas, Angelika Borkowetz, Steffen Löck, Mechthild Krause, Esther G.C. Troost

**Affiliations:** ^1^ Department of Radiotherapy and Radiation Oncology, Faculty of Medicine and University Hospital Carl Gustav Carus TUD Dresden University of Technology Dresden Germany; ^2^ Department of Nuclear Medicine, Faculty of Medicine and University Hospital Carl Gustav Carus TUD Dresden University of Technology Dresden Germany; ^3^ Department of Urology, Faculty of Medicine and University Hospital Carl Gustav Carus TUD Dresden University of Technology Dresden Germany; ^4^ National Center for Tumor Diseases (NCT), NCT/UCC Dresden, A Partnership Between DKFZ, Faculty of Medicine and University Hospital Carl Gustav Carus TUD Dresden University of Technology, and Helmholtz‐Zentrum Dresden‐Rossendorf (HZDR) Dresden Germany; ^5^ OncoRay – National Center for Radiation Research in Oncology, Faculty of Medicine and University Hospital Carl Gustav Carus TUD Dresden University of Technology, Helmholtz‐Zentrum Dresden‐Rossendorf Dresden Germany; ^6^ Helmholtz‐Zentrum Dresden‐Rossendorf Institute of Radiooncology ‐ OncoRay Dresden Germany; ^7^ German Cancer Consortium (DKTK), Partner Site Dresden, and German Cancer Research Center (DKFZ) Heidelberg Germany

**Keywords:** stereotactic body radiotherapy, castration‐resistant prostate cancer, prostate‐specific membrane antigen, positron emission tomography/computed tomography, randomised controlled trial, progression‐directed therapy, oligoprogression, metastasis‐directed therapy, progression‐free survival

## Abstract

**Objective:**

To prove the oncological benefit of ablative radiotherapy in patients with up to five metastases from castration‐resistant prostate cancer (CRPC) a single‐centre randomised trial was initiated.

**Patients and Methods:**

This monocentric, randomised, phase II clinical trial enrolled patients with up to five prostate‐specific membrane antigen‐positive bone or lymph node metastases developing prostate‐specific antigen (PSA) progression during androgen deprivation (ADT) or ADT and androgen‐receptor targeted therapy. Participants were randomised (2:1) to receive metastasis‐directed therapy (MDT) or observation (OBS) without changing systemic therapy. The primary endpoint was the proportion of patients having PSA progression within 1 year, with statistical analyses conducted using intention‐to‐treat principles. Here, results of a planned interim analysis of the primary endpoint are reported.

**Results:**

A total of 30 patients (12 in the observation arm and 18 in the MDT arm) were enrolled, PSA progression within 1 year occurred in 44% of the MDT group vs 75% in the OBS group (*P* = 0.14, not significant). The median time to PSA progression was significantly longer in the MDT arm (12.4 months) compared to the OBS arm (2.9 months, *P* = 0.03). The pre‐defined criteria to discontinue the study were not met. Limitations include the single‐centre design and small sample size at interim analysis.

**Conclusion:**

This pre‐planned interim analysis of the primary endpoint did not meet the discontinuation criteria of the study protocol, suggesting that MDT in oligometastatic CRPC may extend the time to PSA progression without immediate change of systemic therapy. The continuation of the study in a multicentre setting is planned (Institutional funding by the TU Dresden, ClinicalTrials.gov identifier: NCT04141709).

**Abbreviations**
ADT: androgen deprivation therapy
ARTA: androgen receptor targeted agents
CRPC: castration‐resistant prostate cancer
MDT: metastasis‐directed therapy
OBS: observation
Oli‐CR‐P: ‘Local Ablative Radiotherapy for Oligoprogressive Castration Resistant Prostate Cancer’ clinical trial
PET: positron emission tomography
PSMA: prostate‐specific membrane antigen
(SB)RT: (stereotactic body) radiotherapy

## Introduction

Prostate‐specific antigen (PSA) progression is commonly observed after primary curative treatment in patients with prostate cancer. Androgen deprivation therapy (ADT) is a mainstay of oncological treatment in this situation, especially in the presence of metastatic disease [1]. When PSA is increasing during ADT despite suppressed testosterone levels in the blood, the patient is in a castration resistant state [1, 2].

According to international guidelines, the standard treatment options for patients with metastatic, castration‐resistant, asymptomatic or mildly symptomatic and progressive disease include treatment with androgen receptor targeted agents (ARTA), or docetaxel. These palliative treatment options were able to demonstrate a survival benefit between 2 and 5 months compared to any standard therapy in phase III clinical trials [1]. Noteworthy, the optimal time point to introduce next line systemic treatment in the early castration‐resistant situation has not yet been determined. Three clinical trials (PROSPER [ClinicalTrials.gov identifier: NCT02003924], SPARTAN [NCT01946204], and ARAMIS [NCT02200614]) compared ARTA to placebo in the ‘non‐metastatic’ prostate cancer and showed a significant and relevant improvement of clinically relevant endpoints [3, 4, 5]. In these studies the median PSA at inclusion was between 7.8 and 11.1 ng/mL [1]. The metastases‐free survival of ARTA was not significantly different in subgroup analyses when PSA at baseline was above median compared to below baseline. These effective treatments come with clinically significant cardiovascular and neurocognitive side effects especially in an elderly population [6, 7]. In later stages a treatment with a poly‐(ADP‐ribose)‐polymerase (PARP) inhibitor (and ARTA) or prostate‐specific membrane antigen (PSMA) radioligands is established [1].

Prostate‐specific membrane antigen positron emission tomography (PSMA‐PET) is a powerful method for functional staging of prostate cancer in both the hormone sensitive and the castration‐resistant state [8, 9]. In a clinical study of Fendler et al. [10] patients with castration‐resistant prostate cancer (CRPC) and no sign of metastases on conventional anatomical imaging, PSMA‐PET‐positive lesions were regularly detected. Approximately 26% of patients had no metastatic lesions (e.g., a local recurrence or no sign of tumour) and 46% had multiple or disseminated metastases. Another 29% had up to three oligoprogressive metastatic lesions, potentially amendable to metastasis‐directed therapy (MDT). Therefore PSMA‐PET hybrid imaging is recommended by the European Organisation for the Research and Treatment of Cancer (EORTC) imaging group to select candidate patients for clinical trials in early CRPC [11].

Local ablative therapy of oligometastatic disease is emerging, of which stereotactic body radiotherapy (SBRT) is one of the options. The safety and efficacy of SBRT has been widely established for the treatment of metastases in the central nervous system, lungs, bones, liver and lymph nodes, with local control rates typically >90% after 2 years [12, 13]. In metastatic prostate cancer the concept of MDT is established in several clinical conditions [14, 15, 16, 17, 18]. Retrospective and non‐randomised prospective studies were able to show that MDT of oligoprogressive lesions in prostate cancer might postpone transition to next‐line systemic therapy [14, 19, 20, 21]. Meanwhile first results of randomised clinical trials adding MDT to ARTA at progression were published [15, 16, 17, 22].

Metastasis‐directed therapy is delivered in most cases as SBRT. If there is an overlap with previous RT or if there are limiting organs at risk, the treatment can be delivered as fractionated RT. Previous analysis of a prospective trial showed no difference in local control after MDT given as SBRT in three fractions or conventional fractionated RT in 25 fractions [14, 23].

The aim of this study was to investigate the efficacy of MDT without immediate change of systemic therapy compared to continuation of established ADT or ADT + ARTA in patients with oligometastatic CRPC. The biological rationale for MDT in oligoprogressive CRPC may reflect spatially limited clonal resistance amenable to local ablation, thereby preserving systemic therapy efficacy. The primary endpoint was defined as the proportion of patients with PSA progression (defined as nadir after study inclusion +2 ng/mL) 1 year after randomisation. Here, we report on the planned interim analysis of the primary endpoint after the first 30 patients. Secondary endpoints will be analysed after end of recruitment.

## Patients and Methods

### Clinical Trial Protocol

The randomised ‘Local Ablative Radiotherapy for **Oli**goprogressive **C**astration **R**esistant **P**rostate Cancer’ clinical trial (Oli‐CR‐P; ClinicalTrials.gov identifier: NCT04141709) is an ongoing study to evaluate the effectiveness of local ablative RT as MDT compared to observation in patients with PSMA‐PET/CT identified oligoprogressive CRPC, focusing on delaying PSA progression.

The aim of this single‐centre randomised, prospective phase II intervention study is to investigate the effectiveness of local ablative RT as MDT in patients with oligometastases of CRPC after curative primary treatment. The effectiveness is to be tested in comparison with an observation group, in which systemic therapy remained unchanged until PSA progression (defined as PSA nadir after randomisation +2 ng/mL). The locally ablative RT is given either as SBRT or as conventionally fractionated RT. The local Ethics Committee approved the protocol.

### Patients

**Fig. 1:**
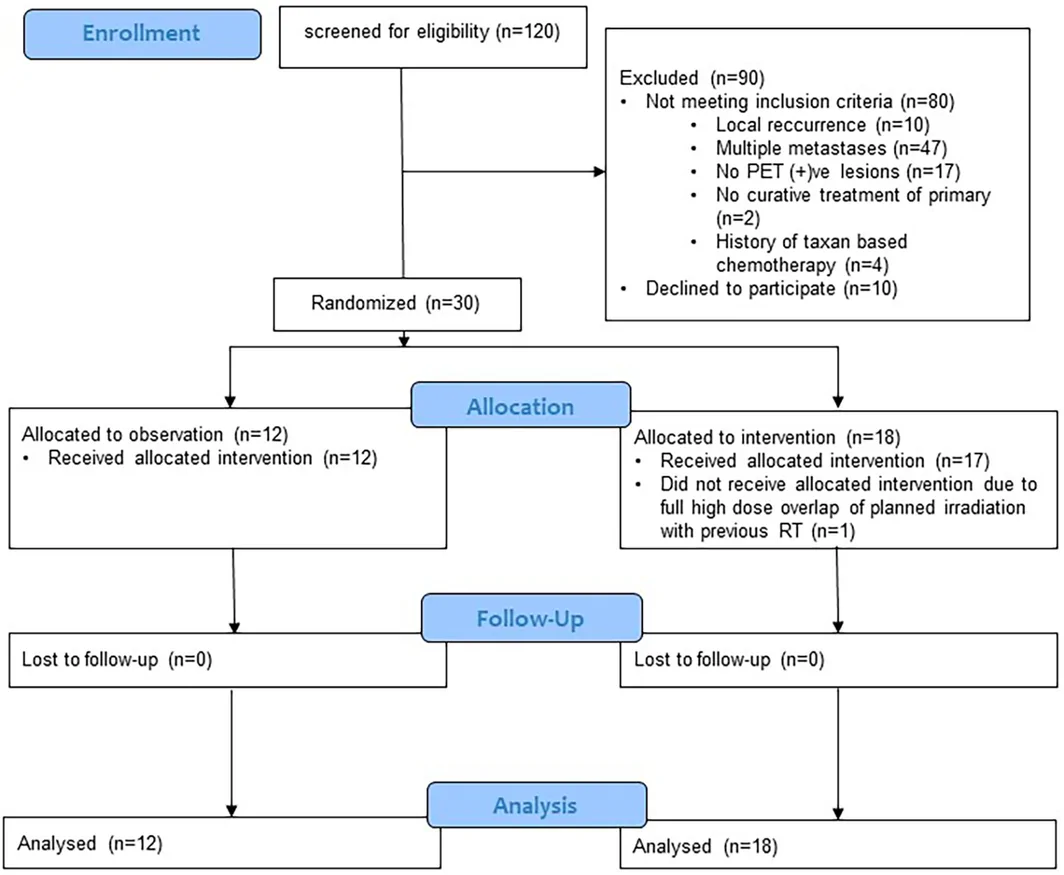
The Consolidated Standards of Reporting Trials (CONSORT) diagram of the Oli‐CR‐P clinical trial.

Patients with PSA progression (defined as three consecutively rising PSA values >4 weeks apart) and castrate levels of testosterone after primary local therapy had PSMA‐PET hybrid imaging within 6 weeks before study inclusion according to local clinical standards (^68^Ga‐PSMA [*n* = 19] or ^18^F‐PSMA [*n* = 11]) and were screened for study participation (Fig. 1). Eligible patients had ADT or ADT + ARTA and up to five PSMA‐positive bone or lymph node metastases. All patients were discussed in a multidisciplinary tumour board. Patients fulfilling the inclusion and exclusion criteria were approached to participate in the study (Table 1).

**Table 1:** Inclusion and exclusion criteria of the Oli‐CR‐P clinical trial.

Inclusion criteria:
Patient in good general condition (ECOG PS 0–1)
Histologically confirmed prostate carcinoma
History of definitive local therapy, e.g., radical prostatectomy or primary RT (also after neo‐/adjuvant ADT, after postoperative RT)
PSA progression during ADT (defined as three consecutively rising PSA values >4 weeks apart and testosterone in the castration range < 50 ng/dL or <1.73 nmol/L)
Minimum duration of ADT 6 months prior to study inclusion
Complete staging (maximum 6 weeks old), preferably using PET hybrid imaging with PSMA‐PET tracer
Evidence of up to five individual active or progressive metastases that are amenable to local ablative RT (histological confirmation of the metastases was not required)
No parallel participation in other clinical therapy studies up to 4 weeks before and after MDT
Individual case discussion in an interdisciplinary tumour board
Ability of the patient to give informed consent
Exclusion criteria:
Serious concomitant disease that limits life expectancy to <5 years according to the physician's assessment
PSA level >20 ng/mL, testosterone level >50 ng/dL (or >1.73 nmoL/L)
Visceral metastases
Lack of compliance
Lack of capacity of the patient to give consent
Previous chemotherapy containing taxane

ECOG PS, Eastern Cooperative Oncology Group Performance Status.

### Randomisation

Eligible patients who gave their written informed consent to participate in the study were randomised 2:1 to the intervention arm (MDT) or to the observation arm (OBS). Randomisation was performed electronically using the institutional study database (RadPlanBio; a German Cancer Consortium [DKTK] platform) [24]. Stratification criteria were the type of ongoing systemic therapy (ADT vs ADT + ARTA), number of metastases (one vs two–five metastases) and type of metastasis (lymph node ± bone vs bone metastasis only).

After randomisation, the current systemic therapy remained unchanged in both treatment arms.

### Radiation Therapy

All patients in the MDT arm were treated with a metastasis‐directed local ablative external beam RT, either as conventionally fractionated RT with 2 Gy/fraction up to a total dose of 50 Gy or as SBRT 10 Gy/fraction up to a total dose of 30 Gy. Metastases with a gross tumour volume of ≤25 mL in the planning CT scan were eligible to be treated with both, hypofractionated SBRT or conventional, fractionation schedules. The decision for the fractionation schedule was left to the treating physician, taking into account the localisation of organs at risk and any previous RT. Both schedules are considered isoeffective in terms of local control [23]. A comparison of both schedules is not foreseen due to low numbers of recruited patients. Details regarding RT planning and delivery have previously been published [25].

### Follow‐Up

The PSA value and the clinical status was recorded during study visits, scheduled 6 weeks, 3, 6, 9, 12, 18 and 24 months after randomisation. To assess the secondary endpoints, the follow‐up continued until death, even after the primary endpoint had been reached, or until 24 months after the last patient inclusion. Regular imaging during follow up was not scheduled. In case of PSA progression, PSMA‐PET imaging for staging purposes was recommended, preferably identical to the imaging modality at study inclusion. New locally ablative treatment options and a change of the systemic therapy were discussed in the multidisciplinary tumour board.

### Clinical Endpoint and Statistical Considerations

The primary objective of the study was to prospectively evaluate the time to PSA progression following MDT of bone or lymph node metastases (one to five metastases) in patients with CRPC. Efficacy was measured as the rate of patients with PSA progression 1 year after randomisation. An event was defined as a PSA increase of ≥2 ng/mL above nadir after randomisation.

The alternative hypothesis to be tested was that MDT could delay the time to subsequent PSA progression, that is, that the fraction of patients with PSA progression in the MDT group was lower compared to the OBS group. The null hypothesis was that the rate was equal at 1 year. Based on previously published retrospective data [19], the required number of patients was calculated using the following two assumptions: Without MDT, the proportion of patients with further PSA progression after 1 year is 90% and with MDT to all PET‐positive metastases, the proportion of patients with a PSA progression after 1 year is 50%.

In a two‐sided test of ratios (Fisher's exact test) with normal approximation and continuity correction, an alpha of 0.049, 1‐beta of 0.8 results in a necessary number of 57 analysable patients. Taking 10% drop‐out into account, 66 patients (22 in the OBS and 44 in the MDT arm) need to be recruited. The calculation was performed with PASS 14.0.8 (NCSS LLC, Kaysville, UT, USA).

A planned interim analysis of the primary endpoint after recruitment of 30 patients was introduced in Amendment 1 of the study protocol. The study would be discontinued with a negative result if the difference in the proportion of patients with PSA progression in both arms was <10%. The study would be terminated with a positive result if the difference between the two arms regarding the primary endpoint was statistically highly significant (*P* < 0.001). The significance level alpha was split between the interim analysis (α= 0.001) and the main analysis (α= 0.049). If the result of the interim analysis did not meet any discontinuation criteria, the study was a forehand planned to be continued as a multicentre study.

As secondary analysis, time‐to‐event curves were calculated by the Kaplan–Meier method and both arms were compared by log‐rank test (IBM SPSS Statistics for Windows (Version 30.0); IBM Corp., Armonk, NY, USA).

## Results

Between November 2019 and July 2023, 120 patients were screened (Fig. 1), of which 40 were eligible, and 10 patients declined randomisation. Thus, 30 patients consented to participate in the clinical study and were randomised 2:1, resulting in 12 patients in the OBS arm and 18 patients in the MDT arm.

Patients’ characteristics are shown in Table 2. In the MDT arm, all but one patient (with one lesion overlapping with previous RT volume) received MDT. In details, of 41 treated metastases, 21 metastases were conventionally fractionated, 20 metastases were treated with SBRT in three fractions.

**Table 2:** Patient and treatment characteristics (MDT).

Variable	MDT arm (*n* = 18)	OBS arm (*n* = 12)
Age at randomisation, years, median (IQR)	69.5 (65.5–76.3)	71.5 (67.3–73.8)
Initial PSA level, ng/mL, median (IQR)	23.9 (11.9–54.4)	11.3 (8.9–50.0)
Initial ISUP score, *n* (%)		
ISUP 1	1 (6)	0
ISUP 2	0	2 (17)
ISUP 3	2 (11)	5 (42)
ISUP 4	8 (44)	4 (33)
ISUP 5	7 (39)	1 (8)
NCCN risk (initial), *n* (%)		
Low or intermediate	2 (11)	5 (42)
High	2 (11)	4 (33)
Very high	10 (56)	2 (17)
N+/M+	4 (22)	1 (8)
Initial treatment, *n* (%)		
Radical prostatectomy	15 (83)	10 (83)
Postoperative RT	7 (39)	5 (42)
Primary RT	3 (17)	2 (17)
Time from primary diagnosis, months, median (IQR)	59 (40.8–94.0)	89 (46.5–120)
Time on ADT, months, median (IQR)	33 (28–45)	33 (19–45)
Systemic therapy, *n* (%)		
ADT alone	13 (72)	9 (75)
ADT + ARTA	5 (28)	3 (25)
PSA at inclusion, ng/mL, median (IQR)	3.6 (1.8–9.4)	2.7 (1.9–4.1)
PSA doubling time, months, median (IQR)	2.6 (1.7–11.1)	3.8 (2.3–12.9)
Number of lesions per patient, *n* (%)		
*n* = 1	9 (50)	6 (50)
*n* = 2	1 (6)	3 (25)
*n* = 3	3 (17)	1 (8)
*n* = 4	3 (17)	2 (17)
*n* = 5	2 (11)	0
Type of lesions, *n* (%)		
Lymph node (N1/M1a)	6 (33)	5 (42)
Bone (M1b)	8 (44)	6 (50)
Lymph node and bone	4 (22)	1 (8)
Treatment schedule (per lesion, *n* = 42), *n* lesions		
50 Gy/2 Gy	21	
30 Gy/10 Gy	20	
Not treated	1	

IQR, interquartile range; ISUP, International Society of Urological Pathology; NCCN, National Comprehensive Cancer Network.

The median follow‐up at the time of the planned interim analysis (18 February 2025) was 29.9 months. No patients had PSMA‐PET imaging before meeting the primary endpoint. After 1 year, PSA‐progression occurred in 44% of the patients treated with MDT compared to 75% continuing their systemic therapy only (*P* = 0.14, Fisher's exact test). As none of the discontinuation criteria for the primary endpoint were met, the trial was continued as multicentre study.

**Fig. 2:**
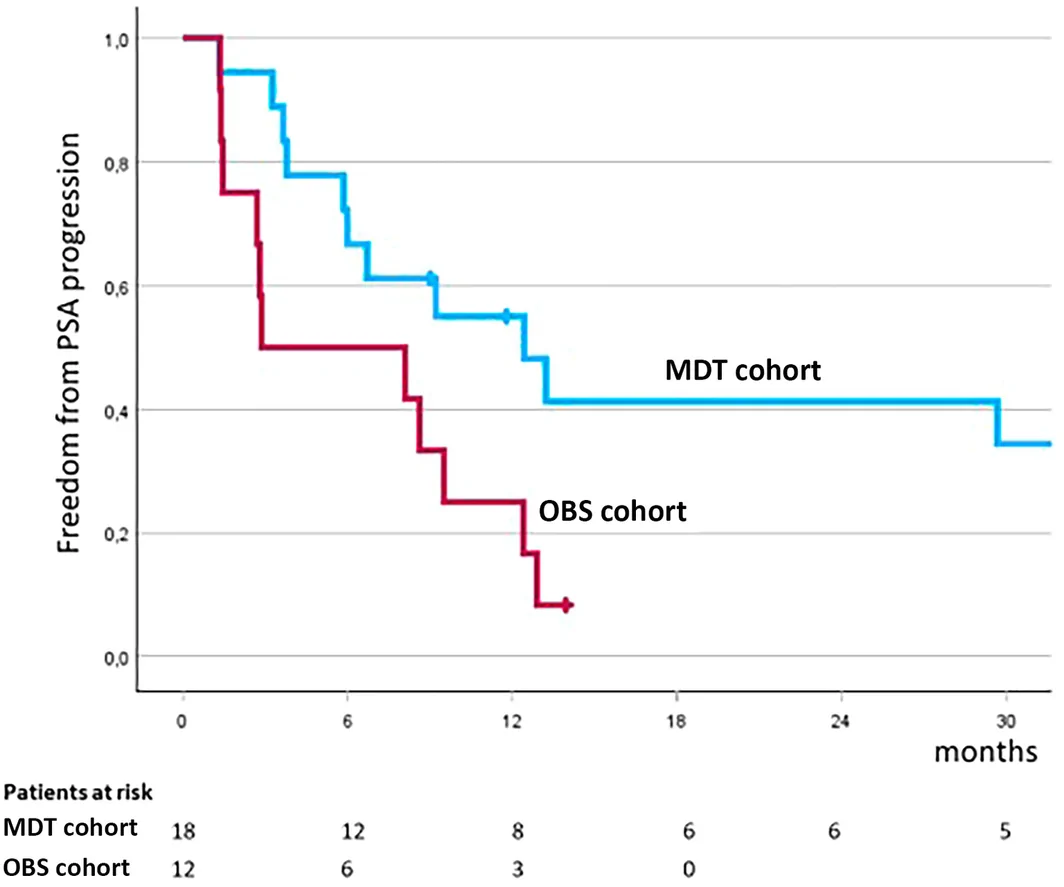
Kaplan–Meier curves for freedom from PSA progression in the OBS cohort (red line) vs MDT cohort (blue line). The difference is statistically significant (*P* = 0.03, log‐rank).

The median time to PSA progression was 12.4 months (95% CI 5.5–19.4 months) for the MDT cohort and 2.9 months (95% CI 0–11.9 months) for the OBS cohort (Fig. 2, *P* = 0.03, log‐rank).

## Discussion

We report on the planned interim analysis of a prospective randomised clinical trial of MDT for oligometastatic CRPC after curative primary therapy. No PSA progression occurred in 56% of the MDT patients compared to 25% of the patients in the OBS cohort 1 year after therapy. To our knowledge this is the first prospective randomised study for MDT in CRPC without immediate change of systemic therapy.

A clinically relevant proportion of participants in the MDT arm benefitted from the locally ablative therapy, thus allowing to continue established ADT (± ARTA) and to delay change of systemic therapy. Postponing toxicities of additional systemic therapy may reflect the wish of patients, as any additional therapy comes with relevant risk for severe complications (e.g., major cardiovascular events or neuro‐cognitive impairment) [6, 7, 26].

The results are promising, as one in three screened patients had an oligoprogressive situation amendable for MDT. A similar rate of patients amendable to MDT was found in a prospective study of Fendler et al. [10]. In 58 of 200 patients with non‐metastatic CRPC in conventional imaging, uni‐ or oligo‐focal disease was detected with PSMA‐PET imaging. Pan et al. [27] found PSMA‐positive oligometastases in 47 of 74 patients progressing early during ADT (PSA level <2 ng/mL).

Prostate‐specific antigen‐progression was selected as primary endpoint for this phase II clinical trial as recommended by the Prostate Cancer Clinical Trials Working Group 3 [2], in order to evaluate the clinical activity of MDT in patients with oligometastatic CRPC. This definition has been adopted similarly by other more recent clinical trials, e.g., ENZA‐p (NCT04419402) [28]. If this effect translates to improvement of clinically relevant endpoints (i.e., time to symptomatic progression, time to further escalation of systemic therapy, or overall survival), this need to be evaluated in a larger randomised study with longer follow up [29].

The time to PSA‐progression of 3 months in the OBS group of our study compares well to the enzalutamide‐only arm (7.8 months) in the ENZA‐p study [28]. Likewise, a median time to PSA‐progression of 12.4 months with MDT, as reported here, corresponds well to our retrospective data that formed the base for the trial (i.e., 15.7 months) [19].

In a non‐randomised prospective study, 29 of 47 patients underwent MDT. Nine patients (28%) receiving MDT had progression after a median follow up 21 months. Of 18 patients not receiving MDT 11 (61%) progressed during follow up [27].

In a first randomised multicentre trial (ARTO trial; NCT03449719), 157 patients with oligometastatic CRPC were included [15]. All patients had escalation of systemic therapy with abiraterone and were randomised to MDT to all metastatic lesions. In contrast to the Oli‐CR‐P trial, only a minority of patients were staged with PSMA‐PET imaging. A statistically significant improvement of biochemical response was reported for MDT and abiraterone (92%) compared to abiraterone alone (68%).

In the multicentre GROUQ‐PCS 9 phase II clinical trial (NCT02685397) 102 ARTA‐naïve patients with oligometastatic CRPC were randomised to MDT additionally to ADT + enzalutamide [22]. The trial was stopped early as ARTA became standard of care for metastatic hormone‐sensitive prostate cancer. The main differences to the OLI‐CR‐P trial were the conventional staging, the immediate transition to a next‐line systemic therapy and the heterogeneous dose concepts for MDT. The addition of MDT to ADT + enzalutamide significantly improved the median radiographic progression‐free survival compared to ADT + enzalutamide alone (from 2.3 to 4.6 years; hazard ratio 0.48, 95% CI 0.27–0.86; *P* = 0.014).

The strength of the study are the stringent inclusion criteria, the assessment of all subsequent patients at our centre, the implementation of PSMA‐PET hybrid imaging, MDT to all PET‐positive metastases and the two uniform RT schedules for MDT. Limitations of the study include the single‐centre design, the prolonged recruitment period caused by the coronavirus disease 2019 (COVID‐19) pandemic and the introduction of ARTA as standard of care, which patients did not receive at the beginning of the study. Further limitations are the lack of regular imaging during follow‐up independently of PSA values and the small number of patients reported in this interim analysis.

Metastasis‐directed therapy in PSMA‐PET staged oligoprogressive CRPC is promising, even without immediate change of systemic treatment. In this planned interim analysis, the addition of MDT lead to a prolonged time to PSA progression at 1 year in a relevant subset of patients. As the pre‐defined criteria for discontinuation of the study were not met in this interim analysis, recruitment continues in the setting of a multicentre study with a target of 66 patients.

## Disclosure of Interests

None declared.

## Funding

The authors have nothing to report.

## Data Availability

The data that support the findings of this study are available from the corresponding author upon reasonable request.
